# Game Species Management and Ecosystem Health: Leveraging Vulture Scavenging to Improve Carcass Disposal and Health Risk Reduction

**DOI:** 10.3390/ani15050732

**Published:** 2025-03-04

**Authors:** Inmaculada Navarro, Raquel Castillo-Contreras

**Affiliations:** Department of Research, Fundación Artemisan, 13001 Ciudad Real, Spain; raquel.castillo@fundacionartemisan.com

**Keywords:** conservation, ecosystem services, health, hunting management, modeling, scavenger birds, photo trap, vultures

## Abstract

Avian scavengers, particularly vultures, play a crucial role in ecosystem health by efficiently consuming carcasses, thereby reducing pathogen abundance and limiting disease transmission to wildlife, livestock, and humans. In addition to the indispensable role of vultures, they are a particularly threatened group of birds. This study investigates the environmental factors that optimize this ecosystem service by examining the scavenging dynamics of vultures and other species at deer carcasses in a hunting area in Sierra Madrona, Ciudad Real, Spain. Carcasses were placed in habitats with different vegetation densities (open vs. dense) and altitudes (high vs. low) and were monitored for 30 days using camera traps. Data on scavenger diversity, arrival times, and carcass persistence were analyzed using Bayesian multilevel models. Results reveal that vegetation density and altitude significantly influence vulture arrival times and carcass duration, with dense vegetation and low altitudes delaying scavenger access. These findings provide actionable insights for game management to enhance vulture conservation and improve both public and ecosystem health through timely and effective carcass removal.

## Simple Summary

Vultures play a crucial role in ecosystem health by removing carcasses and reducing disease transmission. However, they are a highly vulnerable bird group. This study examines the environmental factors influencing scavenging dynamics in a hunting area in Sierra Madrona, Spain. Deer carcasses were placed in habitats with varying vegetation density and altitude and monitored for 30 days using camera traps. Results indicate that dense vegetation and lower altitudes delay vulture arrival and carcass consumption. These findings provide valuable insights for game management to enhance vulture conservation and promote ecosystem and public health.

## 1. Introduction

The role of vultures as keystone scavengers in ecosystems involves maintaining ecological balance and mitigating the spread of diseases. By efficiently disposing of animal carcasses, vultures significantly reduce the risk of pathogen transmission that could otherwise infect wildlife, livestock, and even humans [1]. Their acidic stomachs are uniquely adapted to neutralize harmful bacteria, including *Bacillus anthracis*, *Clostridium botulinum*, and *Salmonella* spp., which can pose significant health risks in environments where carcass accumulation is unmanaged [2,3]. Beyond their preventative role in pathogen transmission, the sanitary function has considerable economic implications. The decline or disappearance of these species in certain regions has led to increased costs in animal waste management, as local authorities must implement artificial methods such as the incineration or burial of carcasses to prevent public health issues [4]. In this regard, vultures are regarded as natural allies in environmental and sanitary management, contributing to the sustainability of livestock and wildlife systems [5]. This critical ecosystem service underscores the importance of preserving vulture populations, which have been severely impacted by anthropogenic threats such as poisoning, habitat loss, and food scarcity, the latter of which has been exacerbated by factors such as sanitary restrictions [6,7].

Game species’ management, particularly through controlled hunting, has emerged as a vital tool for regulating populations of large ungulates, particularly in developed countries. Species such as wild boar (*Sus scrofa*) and red deer (*Cervus elaphus*) often experience uncontrolled population growth due to reduced predation pressure and favorable environmental conditions [8]. Overabundant ungulate populations can lead to severe ecological consequences, including habitat degradation, crop damage, and increased risk of disease transmission, such as bovine tuberculosis (bTB) and African swine fever (ASF) [9,10,11]. Hunting not only helps to control ungulate numbers but also generates carcasses that, if managed appropriately, can benefit scavenger species like vultures, thereby enhancing ecosystem health [12]. Furthermore, the decline of extensive livestock farming in many regions has reduced the availability of natural food resources for vultures [13], increasing their reliance on carcasses from hunting activities [14]. This shift underscores the need for hunting practices to be conducted with awareness of their role in vulture conservation, ensuring proper carcass management to support these critical scavengers and the ecosystem services they provide [15].

The One Health framework offers a powerful lens for exploring the interconnectedness between wildlife, ecosystem services, and human well-being. This integrative approach recognizes that the health of humans, animals, and ecosystems is interconnected, emphasizing the need for collaborative efforts across disciplines to address challenges such as zoonotic disease outbreaks and biodiversity loss [16]. In this context, the role of vultures as natural carcass disposal agents aligns closely with the objectives of One Health, offering an ecologically sustainable solution to disease control and waste management [17]. Vultures, with their unique physiological adaptations, rapidly consume carcasses and neutralize harmful pathogens, reducing the potential for diseases to spread within wildlife–livestock–human interfaces [18]. Therefore, it is essential to ensure that carcasses are in the field for as little time as possible to reduce the risk of pathogen proliferation and disease transmission.

Camera trapping, a widely employed non-invasive monitoring technique, has revolutionized the study of wildlife ecology and behavior [19]. By deploying motion-activated cameras in strategic locations, researchers can gather detailed high-resolution data on the presence, activity patterns, and interactions of diverse species without direct disturbance [20]. This method is particularly valuable for studying scavenger communities, as it allows for the continuous observation of carcass utilization events under natural conditions [21,22]. Camera traps provide insights into species composition, arrival times, feeding hierarchies, and competitive interactions, making them an indispensable tool for assessing the ecological roles of scavengers like vultures [22,23]. Moreover, their ability to capture cryptic behavior enhances our understanding of wildlife dynamics in both pristine and human-modified landscapes, contributing to more informed conservation and management strategies [24,25].

This study examines the role of vultures in carcass disposal within the context of game management practices. Other authors have studied the influence of habitat and other factors on the stay of carcasses in the field, as well as the composition of the scavenger community [22,26,27]. By exploring the intersection of scavenger ecology, disease control, and One Health principles, we aim to highlight the potential for leveraging vulture activity to enhance ecosystem health and inform sustainable management strategies.

## 2. Materials and Methods

### 2.1. Study Area

This study was conducted in Sierra Madrona, a mountain chain belonging to Sierra Morena, located on the southwestern border of the Ciudad Real province (Castilla-La Mancha region, Spain). The study was conducted across four distinct habitat types within a hunting estate, covering an area of approximately 14,000 ha. The habitats were selected based on vegetation type and altitude, as these factors were anticipated to influence the scavenging behavior of vultures [22]. Two main habitat categories were selected as follows:

- Habitat without dense vegetation (open terrain).
- Habitat with dense vegetation.

Each habitat category was further divided into two categories based on altitude as follows:

- High altitude (>1000 m).
- Low altitude (<600 m).

**Figure 1:**
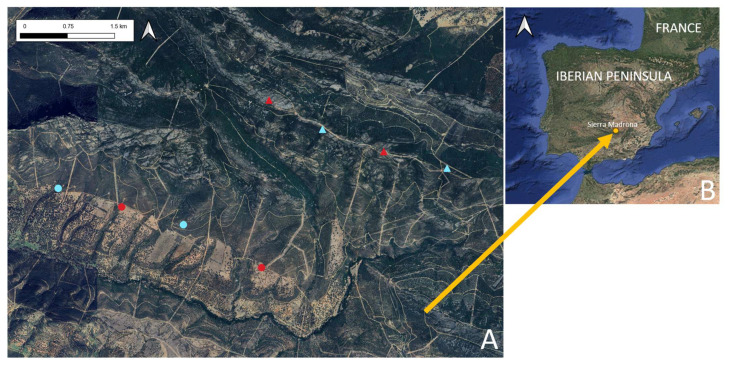
(**A**) Locations of the camera traps. Triangles: high altitude; circles: low altitude; red: open habitat; blue: dense vegetation. (**B**) Location of Sierra Madrona. Source: Google Satellite.

**Figure 2:**
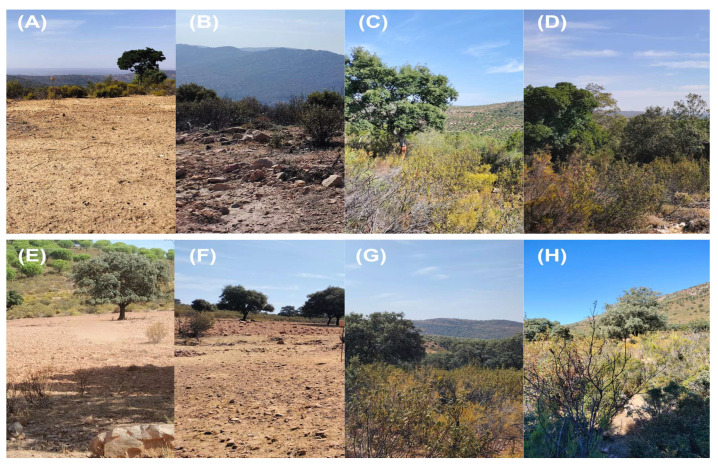
Images of the four habitat types studied, including “Open and high altitude” (**A**,**B**); “Dense and high altitude” (**C**,**D**); “Open and low altitude” (**E**,**F**); and “Dense and low altitude” (**G**,**H**).

Thus, a total of eight sampling points were established for camera placement (Figure 1), with each habitat type being replicated at both high and low altitudes. The points were selected to ensure the homogeneity of conditions (e.g., minimal human presence and similar vegetation). The points were separated from one another by a minimum of 1 km to maximize their independence [28]. The locations of the camera traps were as follows:

Open habitats were free of tree and scrub vegetation, and the grass present covered less than 10% of the ground (Figure 2A,B,E,F). The dense plots were characterized by dense scrub, predominantly rockrose (*Cistus ladanifer*), common thyme (*Thynus vulgaris*) and rosemary (*Salvia rosmarinus*), in addition to shrubs, predominantly *Retama sphaerocarpa*, as well as scattered trees of the genus *Quercus* and *Juniperus*. Herbaceous vegetation covered more than 70% of the ground (Figure 2C,D,G,H). Each placement point was monitored for three repetitions, with each repetition lasting 10 days.

### 2.2. Carcass Preparation and Placement

Female red deer (*Cervus elaphus*) carcasses were provided by the hunting management team of the estate. Carcasses were selected to ensure uniformity in size and weight (N = 24, 81.60 ± 14.13 kg), thereby eliminating variations in scavenger attraction due to differences in carcass size. All the carrion was placed early in the morning. The animals were shot the afternoon prior to placement. In total, 3 carcasses were placed for each selected point.

Carcasses were placed at the predefined habitat points. Each carcass placement was carefully chosen to represent the characteristics of the respective habitat (open vs. dense vegetation; high vs. low altitude).

### 2.3. Camera Trap Setup

Browning Dark Ops HD Pro (BTC-6HDPX) camera traps (Birmingham, AL, USA), equipped with motion sensors and night vision capabilities, were utilized for the study. These cameras are highly suitable for wildlife monitoring due to their capacity for clear image capturing in low-light conditions. The cameras detect the ambient temperature, which was also taken into account in this study.

Cameras were strategically installed 5 m from each carcass to maximize the likelihood of capturing images of vultures and other scavengers that approached the carcasses. The cameras were mounted on nearby trees and were placed at angles that would allow for clear visibility of the carcasses while minimizing disturbance to the wildlife. Camera traps were programmed to capture images in bursts of three with a minimum possible delay between triggers (5 s). This ensured that the activity around the carcasses was recorded in detail, capturing the arrival times and behaviors of scavengers [22].

### 2.4. Monitoring and Data Collection

**Figure 3:**
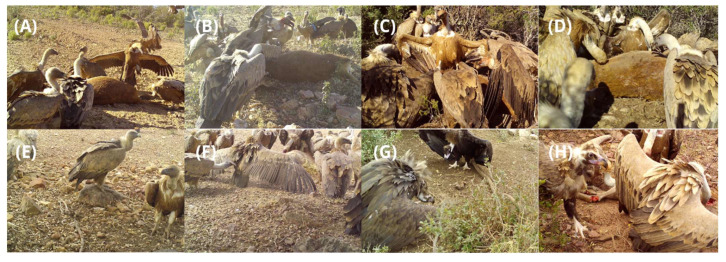
Images of two vulture species recorded in the four habitat types studied: “Open and high altitude” (**A**,**B**), “Dense and high altitude” (**C**,**D**), “Open and low altitude” (**E**,**F**), “Dense and low altitude” (**G**,**H**).

In October 2024, we placed 8 camera traps (1 for each sampling point). We monitored the cameras continuously for a period of 30 days. Each sampling point was continuously monitored for 30 days, checking cameras every 10 days. Each scavenger species observed in the camera footage was identified. The primary focus was on vultures (Figure 3), but other facultative scavengers such as foxes (*Vulpes vulpes*), wild boars (*Sus scrofa*), dogs (*Canis familiaris*), and corvids (*Corvus* spp., *Cyanopica cooki*, *Garrulus glandarius*) were also recorded. A species was classified as a consumer when it was clearly observed feeding on a carcass. The time at which each scavenger species arrived at the carcasses was noted. The carcass was considered consumed once only the bones remained.

### 2.5. Statistical Analyses

To assess whether there were significant differences in carcass persistence time based on vegetation density, we applied the Mann–Whitney U test [29,30], a non-parametric method suitable for comparing independent groups.

To analyze the factors influencing the time carcasses remained in the environment and the arrival times of vultures, we employed Bayesian multilevel models using the brms package [31] in R software version 4.4.1 [32]. The models were designed to assess how different variables, such as habitat, altitude, and temperature, influenced the vulture arrival and carcass stay times while accounting for the potential variability among camera locations in capturing the data.

#### 2.5.1. Vulture Arrival Time Model

To examine the factors influencing the time it took for vultures to arrive at carcasses, we employed a Bayesian multilevel model. This analysis was conducted to assess how various environmental and ecological factors, including habitat type (clear or dense vegetation), altitude (high or low), and other species influenced vulture arrival times. The model also accounted for potential variability across camera locations by incorporating random intercepts for each camera location, thus controlling for spatial differences in vulture presence.

The model was fitted using 4 chains, with each chain running for 4000 iterations and a warm-up period of 2000 iterations. Parallel computation was utilized by setting 4 cores to speed up the sampling process. Additionally, we increased the adapt delta parameter to 0.99 to ensure that the model’s convergence was achieved with a higher level of precision. Convergence diagnostics, such as the potential scale reduction factor (Rhat), effective sample sizes (Bulk ESS and Tail ESS) [33], and Bayesian R2 [34], were used to evaluate the quality of the model fitting and ensure the reliability of parameter estimates.

#### 2.5.2. Carcass Stay Time Model

For the analysis of carcass stay times, we used a Bayesian multilevel model. This model includes fixed effects for vulture arrival times, habitat type (clear or dense vegetation), altitude (high or low), presence or absence of wild boars (*Sus scrofa*), scavenger richness, and temperature, with random intercepts for each camera location to account for the variability in carcass observations across different camera locations.

The model was fitted using 4 chains, with each chain running for 4000 iterations and a warm-up period of 2000 iterations. Parallel computation was utilized by setting 4 cores to speed up the sampling process. Additionally, we increased the adapt delta parameter to 0.99 to ensure that the model’s convergence was achieved with a higher level of precision. Convergence diagnostics, such as the potential scale reduction factor (Rhat), effective sample sizes (Bulk ESS and Tail ESS) [33], and Bayesian R2 [34], were examined to assess the quality of the model fitting and the reliability of the parameter estimates.

## 3. Results

We analyzed 67,510 photos taken during the 30-day study period and detected feeding from two obligate scavengers (*Gyps fulvus* and *Aegypius monachus*) and four facultative scavengers (*Corvus corax*, *Sus scrofa*, *Canis familiaris*, and *Vulpes vulpes*). Out of the 24 carcasses, the wild boar was present in 18 (75%) carcasses, fox in two (8.33%), and raven in three (12.5%). Dogs were found in one (4.16%). Of the 24 carcasses, 18 were completely eliminated, with 13 carcasses disposed of by griffon vultures (mean stay time = 24.25 ± 33.10 h) and 5 carcasses disposed of by wild boars (mean stay time = 157.95 ± 22.52 h).

In open habitats, the average time the carcass spent in the field (i.e., the time until only bones of the animal are left) was 50.89 ± 75.05 h, while in densely vegetated habitats, the average time was 132.22 ± 71.31 h (U = 31, *p* < 0.05). Where longer carcass stay times have been observed in dense habitats, as well as a longer delay in the arrival of vultures (Table 1).

The Bayesian multilevel models (Table 2) revealed that vegetation and altitude were significant predictors of the timing of vulture arrival, with dense vegetation and high altitude being associated with a later arrival time. The model explained 49% of the variance in vulture arrival time, highlighting the importance of habitat factors. In terms of carcass duration in the field, the variables that positively influenced the length of time a carcass spent in the field were vulture arrival time, dense vegetation, and the presence of wild boars, while high altitude had a negative influence on the length of time a carcass spent in the field, i.e., the higher the altitude, the lower the value of the response variable. The model explained 61% of the variance in carcass stay time.

## 4. Discussion

Our study sheds light on the complex dynamics of scavenger activity and carcass persistence in Mediterranean ecosystems, emphasizing the roles of habitat characteristics and species interactions. The findings have important implications for understanding scavenger hunting-related ecology and developing management practices to support obligate scavengers, such as vultures.

We documented six scavenger species interacting with carcasses, including two obligate (*Gyps fulvus* and *Aegypius monachus*) and four facultative scavengers (*Corvus corax*, *Sus scrofa*, *Canis familiaris*, and *Vulpes vulpes*). Notably, the wild boar (*Sus scrofa*) was the most frequent facultative scavenger, present at 75% of carcasses compared to foxes (8.33%), crows (12.5%), and dogs (4.16%). The high prevalence of wild boars highlights its role as a dominant facultative scavenger in the region, which aligns with previous studies documenting Sus scrofa as an important scavenger in various ecosystems across Europe, including Spain, Poland, and The Netherlands, where it frequently consumes carrion from both wild and domestic animals [35,36,37]. This underscores the potential competition between facultative and obligate scavengers, which could influence the ecosystem services provided by scavengers. Furthermore, the presence of facultative scavengers at carcasses has important implications for disease ecology, as these species are known to act as a potential vectors for the spread of pathogens, such as tuberculosis and rabies, through their scavenging activity and movements across the landscape [5]. The increased interaction of dogs and wild boars with carcasses could amplify pathogen transmission risks, not only for wildlife but also for livestock and human populations, underscoring the need for targeted management strategies [5,38].

The Bayesian multilevel models conducted in this study demonstrate that habitat features play a critical role in determining the arrival time of vultures and the duration of carcasses in the field. Dense vegetation and low-altitude sites delayed vulture arrival, likely due to reduced visibility and accessibility. These factors explained 49% of the variance in vulture arrival time, indicating the importance of habitat structure in shaping scavenger behavior. Similarly, carcass persistence was strongly influenced by vegetation and altitude, as well as by the arrival time of vultures and the presence of wild boars. Carcasses in dense vegetation or visited by wild boars remained longer in the field, while those at higher altitudes exhibited shorter persistence times. These factors accounted for 61% of the variance in carcass stay time.

While facultative scavengers play an important role in carrion detection (eagles, corvids, etc.) [39], in our study, the first carrion detector was the griffon vulture, followed by the wild boar and, finally, the cinereous vulture. This fact may be due to the large number of griffon vultures present in the study area [40]. In this context, in the assumption of a recolonization of the bearded vulture (*Gypaetus barbatus*) in the study area, it can be deduced that the griffon vulture would be the main facilitator to find carrion quickly, as observed in other areas [41]. This suggests that the griffon vulture’s presence could facilitate the bearded vulture’s ability to find food resources more efficiently.

Our findings align with previous knowledge on vulture foraging behavior, reinforcing the importance of habitat structure and interspecific interactions in scavenger dynamics. Notably, our results are consistent with Oliva-Vidal et al. [22], who also highlighted the influence of vegetation and altitude on carcass consumption patterns in Pyrenees. Furthermore, photographic evidence from our study suggests that once a wild boar arrives at a carcass, vultures no longer feed on it. In fact, the footage shows that vultures do not even attempt to approach carcasses already occupied by wild boars, possibly due to avoidance behavior or direct competition. This could indicate that the wild boar plays a dominant role in scavenger hierarchies, effectively excluding vultures from carrion resources, given its high abundance [9]. In this sense, supplementary feeding stations (SFSs) could be a solution in certain circumstances, but these facilities also have their weaknesses, such as the modification of the vultures’ natural behavior [39,42] or the congregation of individuals (and therefore the possibility of spreading diseases such as avian flu) [39]. The optimal solution would therefore be to help the vultures to find the carcasses naturally, leaving them in more accessible areas, as our results indicate.

Lastly, the wild boar appears to significantly alter carcass availability through their behavior. Camera trap images reveal that they frequently move carcasses or carcass remains, often dragging them into dense vegetation or other inaccessible areas. This could further limit vulture access to carrion, as these locations reduce visibility and make it harder for vultures to land and feed. These findings suggest that wild boars may not only outcompete vultures for carrion but also influence carcass decomposition dynamics by modifying their spatial distribution. Understanding these interactions provides new insights into the complex ecological relationships shaping scavenger communities.

### Implications for Conservation

The delayed arrival of vultures in dense vegetation and at low altitudes has significant conservation implications. Obligate scavengers, such as vultures, rely on rapid carcass detection to meet their energetic demands, and delays may reduce their access to food resources. Furthermore, the prolonged persistence of carcasses in dense vegetation may increase the risk of zoonoses, as well as the transmission of other diseases affecting both wildlife and livestock [5,43].

To promote the conservation of vultures and enhance their role as ecosystem service providers, it is essential to optimize carcass availability in the landscape [44]. Specifically, we recommend that hunters and wildlife managers consider habitat characteristics when leaving carcasses in the field after hunting activities, especially after stalk hunts. Carcasses placed in open habitats with good visibility and accessibility are more likely to be located quickly by vultures, thereby reducing the opportunity for facultative scavengers like wild boars to dominate. This approach would favor obligate scavengers, reducing interspecific competition and ensuring that vultures, as specialist consumers, can access resources efficiently.

The findings of this study reinforce the importance of integrating game management into conservation strategies for scavenger species. Open habitats not only enhance the foraging efficiency of vultures but may also reduce human–wildlife conflicts associated with wild boar scavenging by decreasing the risk of disease transmission to humans and livestock, including African swine fever, brucellosis, tuberculosis, and trichinellosis (among others). In addition, limiting carcass access to wild boars may help control their population growth, reducing crop damage, vehicle collisions, and incursions into urbanized areas, which are increasingly problematic as wild boar populations continue to increase [45,46,47,48,49]. Moreover, ensuring that carcasses are accessible to vultures can contribute to their population stability, as food availability remains a key limiting factor for these species in many regions [50,51]. Future conservation efforts should incorporate these insights into habitat and game management practices and scavenger monitoring programs to maintain healthy scavenger populations and the ecosystem functions they support, taking into account both the reduction in extensive livestock farming and the increase in big game hunting [9,13,52].

## 5. Conclusions

In conclusion, our study highlights the intricate interplay between habitat features, species interactions, and scavenger behavior. By aligning conservation efforts with ecological insights, we can mitigate the challenges faced by obligate scavengers and enhance their ecological roles in maintaining healthy ecosystems. In this respect, the role of the hunter is fundamental. We want to emphasize that game carcasses should be placed in the best possible location according to scientific evidence.

Furthermore, our findings underscore the need for a more integrated approach to scavenger conservation and game management. The interactions between obligate and facultative scavengers, particularly the competitive dominance of wild boars, highlight the importance of strategic carcass placement to ensure efficient resource use by vultures. Future research should explore long-term population dynamics and behavioral adaptations of scavengers in response to habitat modifications, as well as assess the broader ecological consequences of facultative scavenger dominance. By implementing science-based management strategies, we can foster a more balanced scavenger community, ultimately strengthening the ecological services they provide.

## Figures and Tables

**Table 1:** Descriptive analyses (mean and standard deviation) broken down by habitat type (open and high altitude; dense and high altitude; open and low altitude; dense and low altitude). TAFS = Time of Arrival of First Scavenger, TAV = Time of Arrival of Vultures; CST = Carcass stay time.

Habitat Type	Altitude (m)	Temperature (°C)	TAFS (hours)	TAV (hours)	CST (hours)
Open and high altitude	1008.5 ± 28.5	26.5 ± 5.1	3.67 ± 4.01	3.67 ± 4.01	12.17 ± 9.61
Dense and high altitude	1013.5 ± 12.5	24.33 ± 2.05	9.41 ± 8.74	16.75 ± 20.03	166.59 ± 45.46
Open and low altitude	578.5 ± 14.5	19.67 ± 3.04	15.54 ± 13.44	10.31 ± 9.52	89.62 ± 85.06
Dense and low altitude	545.0 ± 43.5	25.83 ± 4.74	13.44 ± 8.07	10.41 ± 7.00	97.87 ± 66.96

**Table 2:** Bayesian models used to assess the effect of some variables in vulture arrival time and carcass stay time. We present the variable coefficients and standard error (SE) for models. Significant 95% CI values (without 0) are highlighted in bold. The percentage of explained deviance of the model (i.e., percentage of the variability explained by each model “BR^2^”) is shown. At convergence, Rhat = 1.

Model	Variables	Coefficient	SE	95% CI	Rhat	BR^2^
*Vulture arrival time*	Camera (random)	0.86	0.48	(−0.04, 2.34)	1.00	49%
	Dense vegetation	0.53	0.39	**(0.76, 2.50)**	1.00	
	High altitude	−0.27	0.54	**(0.28, 0.75)**	1.00	
	Temperature	−0.02	0.99	(−1.48, 2.43)	1.00	
*Carcass stay time*	Camera (random)	1.05	0.65	(−0.07, 2.54)	1.00	61%
	Vulture arrival time	0.39	0.03	**(0.09, 0.20)**	1.00	
	Dense vegetation	1.63	0.97	**(0.49, 3.62)**	1.00	
	High altitude	−0.29	0.50	**(0.06, 1.13)**	1.00	
	Wild boar presence	0.59	0.77	**(0.01, 1.99)**	1.00	
	Temperature	0.21	0.84	(−1.89, 2.01)	1.00	

## Data Availability

The data presented in this study are available in this article and from the corresponding author upon reasonable request.

## References

[1] Markandya A., Taylor T., Longo A., Murty M., Murty S., Dhavala K. (2008). Counting the cost of vulture decline—An appraisal of the human health and other benefits of vultures in India. Ecol. Econ..

[2] Ogada D.L., Keesing F., Virani M.Z. (2012). Dropping dead: Causes and consequences of vulture population declines worldwide. Ann. N. Y. Acad. Sci..

[3] Campbell M.O.N. (2021). Vulture foraging and the chemistry of putrefaction. Critical Research Techniques in Animal and Habitat Ecology: International Examples.

[4] Margalida A., Colomer M.À. (2012). Modelling the effects of sanitary policies on European vulture conservation. Sci. Rep..

[5] Ishwar N.M., Das S. (2024). Economics of conserving endangered birds: The case for Gyps vultures in India. Environ. Dev. Sustain..

[6] Colomer M.A., Margalida A. (2025). Demographic Effects of Sanitary Policies on European Vulture Population Dynamics: A Retrospective Modeling Approach. Ecol. Appl..

[7] McClure C.J., Westrip J.R., Johnson J.A., Schulwitz S.E., Virani M.Z., Davies R., Symes A., Wheatley H., Thorstrom R., Amar A. (2018). State of the world’s raptors: Distributions, threats, and conservation recommendations. Biol. Conserv..

[8] Gortázar C., Fernandez-de-Simon J. (2022). One tool in the box: The role of hunters in mitigating the damages associated to abundant wildlife. Eur. J. Wildl. Res..

[9] Carpio A.J., Apollonio M., Acevedo P. (2021). Wild ungulate overabundance in Europe: Contexts, causes, monitoring and management recommendations. Mamm. Rev..

[10] Linnell J.D., Cretois B., Nilsen E.B., Rolandsen C.M., Solberg E.J., Veiberg V., Kaczensky P., Van Moorter B., Panzacchi M., Rauset G.R. (2020). The challenges and opportunities of coexisting with wild ungulates in the human-dominated landscapes of Europe’s Anthropocene. Biol. Conserv..

[11] De Garine-Wichatitsky M., Miguel E., Kock R., Valls-Fox H., Caron A., Vicente J., Vercauteren K.C., Gortázar C. (2021). The ecology of pathogens transmission at the wildlife-livestock interface: Beyond disease ecology, towards socio-ecological system health. Diseases at the Wildlife-Livestock Interface: Research and Perspectives in a Changing World.

[12] Gonzálvez M., Paniagua J., Jiménez-Martín D., Cano-Terriza D., Castro-Scholten S., Barbero-Moyano J., Jiménez-Ruiz S., García-Bocanegra I. (2023). Monitoring the dynamics of consumption of ungulate game by-products in vulture feeding stations in Iberian Mediterranean ecosystems. Res. Vet. Sci..

[13] Ruiz-Mirazo J. Environmental benefits of extensive livestock farming: Wildfire prevention and beyond. Economic, Social and Environmental Sustainability in Sheep and Goat Production Systems. Proceedings of the International Seminar of the Sub-Network on Production Systems of the FAO-CIHEAM Inter-Regional Cooperative Research and Development Network on Sheep and Goats.

[14] Aguilera-Alcalá N., Arrondo E., Pascual-Rico R., Morales-Reyes Z., Gil-Sánchez J.M., Donázar J.A., Moleón M., Sánchez-Zapata J.A. (2022). The value of transhumance for biodiversity conservation: Vulture foraging in relation to livestock movements. Ambio.

[15] Jackson A.L., Ruxton G.D., Houston D.C. (2008). The effect of social facilitation on foraging success in vultures: A modelling study. Biol. Lett..

[16] Adisasmito W.B., Almuhairi S., Behravesh C.B., Bilivogui P., Bukachi S.A., Casas N., Becerra N.C., Charron D.F., Chaudhary A., One Health High-Level Expert Panel (OHHLEP) (2022). One Health: A new definition for a sustainable and healthy future. PLoS Pathog..

[17] Ottinger M.A., Botha A., Buij R., Coverdale B., Gore M.L., Harrell R.M., Hassell J., Krüger S., McClure C.J.W., Mullinax J.M. (2021). A strategy for conserving Old World vulture populations in the framework of One Health. J. Raptor Res..

[18] Van Den Heever L., Thompson L.J., Bowerman W.W., Smit-Robinson H., Shaffer L.J., Harrell R.M., Ottinger M.A. (2021). Reviewing the role of vultures at the human-wildlife-livestock disease interface: An African perspective. J. Raptor Res..

[19] Delisle Z.J., Flaherty E.A., Nobbe M.R., Wzientek C.M., Swihart R.K. (2021). Next-generation camera trapping: Systematic review of historic trends suggests keys to expanded research applications in ecology and conservation. Front. Ecol. Evol..

[20] Burton A.C., Neilson E., Moreira D., Ladle A., Steenweg R., Fisher J.T., Bayne E., Boutin S. (2015). REVIEW: Wildlife camera trapping: A review and recommendations for linking surveys to ecological processes. J. Appl. Ecol..

[21] Walker M.A., Uribasterra M., Asher V., Getz W.M., Ryan S.J., Ponciano J.M., Blackburn J.K. (2021). Factors influencing scavenger guilds and scavenging efficiency in Southwestern Montana. Sci. Rep..

[22] Oliva-Vidal P., Sebastián-González E., Margalida A. (2022). Scavenging in changing environments: Woody encroachment shapes rural scavenger assemblages in Europe. Oikos.

[23] Herbert A.M., Zollner P.A., Jones L.R., Wahl M.L., Burcham G.N., Kluever B.M., Humberg L.A., Quinby B.M. (2024). Competitive Behaviors Between Black Vultures (*Coragyps atratus*) and Turkey Vultures (*Cathartes aura*) During Scavenging. J. Raptor Res..

[24] Cullen J.A., Attias N., Desbiez A.L., Valle D. (2023). Biologging as an important tool to uncover behaviors of cryptic species: An analysis of giant armadillos (*Priodontes maximus*). PeerJ.

[25] Abrha A.M. (2024). Habitat Use, Breeding Biology, and Effects of Climate Change on Two Endemic Francolins in Ethiopia. Ph.D. Thesis.

[26] Arrondo E., Morales-Reyes Z., Moleón M., Cortés-Avizanda A., Donázar J.A., Sánchez-Zapata J.A. (2019). Rewilding traditional grazing areas affects scavenger assemblages and carcass consumption patterns. Basic Appl. Ecol..

[27] Damiani G., Posillico M. (2024). Alimentación nocturna del buitre lenado Gyps fulvus: Una respuesta a la perturbación humana y a la presencia de mamíferos depredadores. Ardeola.

[28] Morales-Reyes Z., Sánchez-Zapata J.A., Sebastián-González E., Botella F., Carrete M., Moleón M. (2017). Scavenging efficiency and red fox abundance in Mediterranean mountains with and without vultures. Acta Oecol..

[29] Wilcoxon F. (1945). Individual comparisons by ranking methods. Biometr. Bull..

[30] Mann H.B., Whitney D.R. (1947). On a test of whether one of two random variables is stochastically larger than the other. Ann. Math. Stat..

[31] Bürkner P.C. (2017). Bayesian Item Response Modeling in R with brms and Stan. J. Stat. Softw..

[32] R Core Team (2024). R: A Language and Environment for Statistical Computing.

[33] Gelman A., Rubin D.B. (1992). Inference from iterative simulation using multiple sequences. Stat. Sci..

[34] Gelman A., Goodrich B., Gabry J., Vehtari A. (2019). R-squared for Bayesian regression models. Am. Stat..

[35] Selva N. (2004). The role of scavenging in the predator community of Bialowieza Primeval Forest. Ph.D. Thesis.

[36] Herrero J., Irizar I., Laskurain N.A., García-Serrano A., García-González R. (2005). Fruits and roots: Wild boar foods during the cold season in the southwestern Pyrenees. Ital. J. Zool..

[37] Wenting E., Jansen P.A., Pattipeilohy L., Van Lunteren P., Siepel H., Van Langevelde F. (2024). Influence of tree cover on carcass detection and consumption by facultative vertebrate scavengers. Ecol. Evol..

[38] Safford R., Andevski J., Botha A., Bowden C.G., Crockford N., Garbett R., Margalida A., Ramírez I., Shobrak M., Tavares J. (2019). Vulture conservation: The case for urgent action. Bird Conserv. Int..

[39] Cortés-Avizanda A., Blanco G., DeVault T.L., Markandya A., Virani M.Z., Brandt J., Donázar J.A. (2016). Supplementary feeding and endangered avian scavengers: Benefits, caveats, and controversies. Front. Ecol. Environ..

[40] Del Moral J.C., Molina B., Molina B., Nebreda A., Muñoz A.R., Seoane J., Real R., Bustamante J., del Moral J.C. (2022). Supplementary feeding and endangered avian scavengers: Benefits, caveats, and controversies. III Atlas de las Aves en Época de Reproducción en España.

[41] Oliva-Vidal P., Villalba D., Colomer M.À., Margalida A. (2024). Heterospecific visual cues and trophic facilitation processes used by a solitary bone-eating vulture. Ecosphere.

[42] Deygout C., Gault A., Sarrazin F., Bessa-Gomes C. (2009). Modeling the impact of feeding stations on vulture scavenging service efficiency. Ecol. Modell..

[43] Gortazar C., Diez-Delgado I., Barasona J.A., Vicente J., De La Fuente J., Boadella M. (2015). The wild side of disease control at the wildlife-livestock-human interface: A review. Front. Vet. Sci..

[44] Mariyappan M., Rajendran M., Velu S., Johnson A.D., Dinesh G.K., Solaimuthu K., Kaliyappan M., Sankar M. (2023). Ecological role and ecosystem services of birds: A review. Int. J. Environ. Clim..

[45] Bruinderink G.G., Hazebroek E. (1996). Ungulate traffic collisions in Europe. Conserv. Biol..

[46] Schley L., Roper T.J. (2003). Diet of wild boar *Sus scrofa* in Western Europe, with particular reference to consumption of agricultural crops. Mamm. Rev..

[47] Meng X.J., Lindsay D.S., Sriranganathan N. (2009). Wild boars as sources for infectious diseases in livestock and humans. Philos. Trans. R. Soc. Lond. B Biol. Sci..

[48] Massei G., Kindberg J., Licoppe A., Gačić D., Šprem N., Kamler J., Baubet E., Hohmann U., Monaco A., Ozoliņš J. (2015). Wild boar populations up, numbers of hunters down? A review of trends and implications for Europe. Pest Manag. Sci..

[49] Castillo-Contreras R., Carvalho J., Serrano E., Mentaberre G., Fernández-Aguilar X., Colom A., González-Crespo C., Lavín S., López-Olvera J.R. (2018). Urban wild boars prefer fragmented areas with food resources near natural corridors. Sci. Total Environ..

[50] Carrete M., Grande J.M., Tella J.L., Sánchez-Zapata J.A., Donázar J.A., Díaz-Delgado R., Romo A. (2007). Habitat, human pressure, and social behavior: Partialling out factors affecting large-scale territory extinction in an endangered vulture. Biol. Conserv..

[51] Zuberogoitia I., Martínez J.E., González-Oreja J.A., Pérez de Ana J.M., Zabala J. (2019). Factors affecting population regulation of a colonial vulture. IBIS.

[52] Carvalho J., Carpio A., Figueiredo A.M., Fonseca C., Ferreira E., Serrano E., Barja I., Sánchez-Zapata J., Carranza J., Palacios L.B. (2025). Three Decades of Research on Iberian Wild Ungulates: Key Insights and Promising Research Avenues. Mamm. Rev..

